# Attitudes Toward Health Care Virtual Communities of Practice: Survey Among Health Care Workers

**DOI:** 10.2196/15176

**Published:** 2019-12-04

**Authors:** Nicole Yada, Milena Head

**Affiliations:** 1 DeGroote School of Business McMaster University Hamilton, ON Canada

**Keywords:** virtual community of practice, eHealth, digital health, knowledge translation, implementation science, elaboration likelihood model, technology adoption

## Abstract

**Background:**

Virtual communities of practice (VCoPs) have been shown to be an effective means for knowledge and research uptake, but little is known about why health care workers choose to use them. The elaboration likelihood model (ELM) is a theoretical model of persuasion that distinguishes between different routes of information processing that influence attitude formation and change. To date, no research has investigated the antecedents to these processing routes for VCoPs within a health care setting. In understanding these determinants, VCoPs can be appropriately designed to increase their chances of use and value among health care professionals.

**Objective:**

Our aim is to explore how motivation and ability affect attitudes toward using VCoPs for those working in health care.

**Methods:**

Data were collected from 86 health care workers using an online survey at two Canadian health care conferences. Participants were shown a mock VCoP and asked about their perceptions of the online platform and related technologies. The survey instrument was developed based on previously validated scales to measure participants’ ability and motivation toward using a VCoP. Attitudes were assessed both at the beginning and end of the study; intention to use the platform was assessed at the end.

**Results:**

Ability (expertise with CoPs and VCoPs) was found to directly affect intention to use the system (*P*<.001 and *P*=.009, respectively) as was motivation (*P*<.001). Argument quality had the greatest effect on formed attitudes toward VCoPs, regardless of the user’s level of experience (lower expertise: *P*=.04; higher expertise: *P*=.003). Those with higher levels of CoPs expertise were also influenced by peripheral cues of source credibility (*P*=.005 for attitude formation and intention to use the system) and connectedness (*P*=.04 for attitude formation; *P*=.008 for intention to use the system), whereas those with lower levels of CoP expertise were not (*P*>.05). A significant correlation between formed attitude and intention to use the VCoPs system was found for those with higher levels of expertise (*P*<.001).

**Conclusions:**

This research found that both user ability and motivation play an important and positive role in the attitude toward and adoption of health care VCoPs. Unlike previous ELM research, evidence-based arguments were found to be an effective messaging tactic for improving attitudes toward VCoPs for health care professionals with both high and low levels of expertise. Understanding these factors that influence the attitudes of VCoPs can provide insight into how to best design and position such systems to encourage their effective use among health care professionals.

## Introduction

### Motivation and Background

Health care in Canada remains fragmented regarding access and delivery. As a result, there is a growing push to enhance integration across sectors, locations, and providers to improve patient experience [1]. This includes greater recognition of the need to improve information sharing and enhanced communication to meet these needs [1]. One way to achieve these goals is through communities of practice (CoPs), which are increasingly used in health care as a means of advancing knowledge use and creation through collaborative learning [2,3]. A CoP is defined as “a group of people who share a concern, a set of problems, or a passion about a topic, and who deepen their knowledge and expertise in this area by interacting on an ongoing basis” [4].

A CoP becomes a virtual community of practice (VCoP) when its members use information and communication technologies (ICTs) as their primary means for collaboration and communication. Although VCoPs do not exclude face-to-face meetings, ICT help to overcome geographic and organizational barriers, thereby increasing the efficiency of information sharing [5]. In a health care context, VCoPs allow their members to access information relevant to their practice on an as-needed basis [6], facilitate problem solving through active debate and integration of differing perspectives [7], and enable health care providers to efficiently stay up-to-date on the increasing medical evidence base and share newly acquired information with their peers [8]. However, it is important to note that relevant content is necessary to maintain engagement among VCoP members, which can quickly become outdated due to the rapidly changing evidence base and policies present in health care [7]. Additionally, many VCoP members prefer to observe and not participate, whereas some clinicians prefer to communicate only with members of their own specialty, thus limiting the potential to advance interprofessional knowledge [7,9]. Overall, research has demonstrated the effectiveness of these VCoPs for health care quality improvement, but little is known about how and why users choose to adopt technology to support CoPs [9-11]. Further, technology adoption in health care is subject to unique factors and influences [12]. To ensure the successful implementation of such a technology, it is important to understand the factors influencing the decision to use it.

### Theoretical Framework

Understanding the process by which intentions to use a platform such as a VCoP are formed is necessary, and the elaboration likelihood model (ELM) [13] provides a promising conceptual framework through which to study this. The ELM [13] is a model of persuasion that posits that users process given information (or “elaborate”) based on their level of motivation and ability related to the issue at hand, leading to attitude formation and change. When information processing leads to a change in attitude, this is referred to as *persuasion* [14]. Widely validated as a framework in psychology and marketing (eg, [15]), the ELM is considered the foremost influential model used to study attitude formation and change [16].

Persuasion theories are based on the assumption that individuals process the same messages with varying levels of effort [17]. The ELM is a dual-process model that posits two routes of information processing that differ based on extent (more versus less) of processing [18]: (1) the central route, where users are highly motivated or able and are influenced by argument quality, and (2) the peripheral route, where users are not as highly motivated or able and are influenced by more superficial factors, such as the likability of the endorser, source credibility, and message medium. Attitudes formed through the latter process tend to be less stable over time [19].

**Figure 1:**
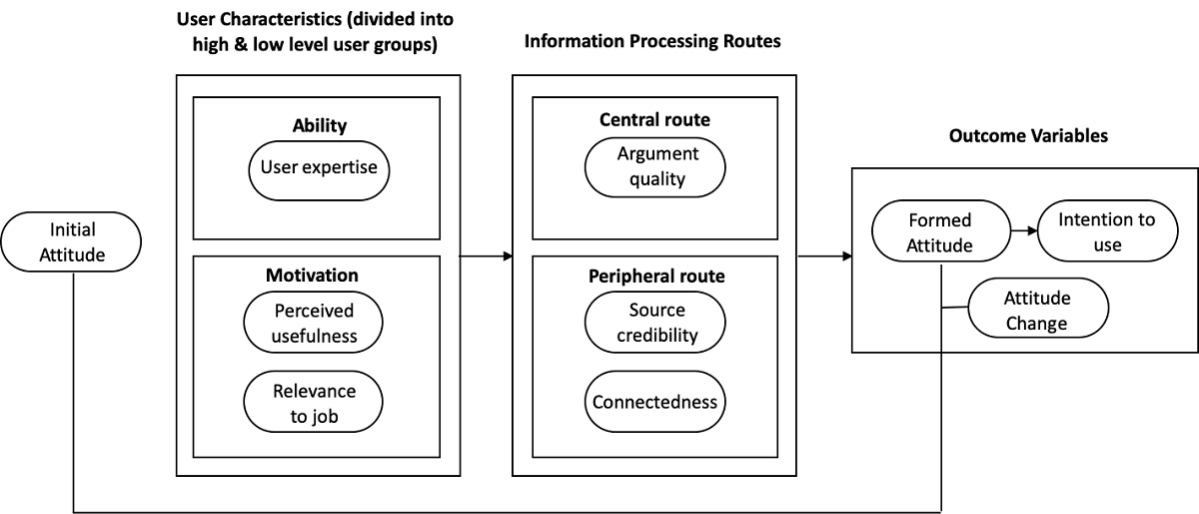
Research model to investigate attitudes and intention to use health care virtual community of practice.

The ELM was used to guide this study. This model suggests that a person’s characteristics of motivation and ability determine the route through which they process information and form attitudes, leading to their intention to use the system [20]. As such, antecedents to VCoP processing routes have been divided into two broad categories: (1) ability (operationalized through four types of user experience or expertise) and (2) motivation to use the technology (operationalized through the constructs of relevance to job and perceived usefulness). The ELM also suggests that differing levels of ability and motivation may influence information processing and outcome variables, such as attitude formation and change as well as intention to use. Thus, we further investigated these antecedents by separating them into high and low levels for each user characteristic. Our research model built off of the ELM framework is shown in Figure 1.

### Ability (User Expertise)

Expertise can refer to domain expertise (ie, understanding of health care) or system expertise (ie, ability to use a VCoP system in general). The focus of this paper is on system expertise because the VCoP target users are health care workers who possess health care domain expertise. According to Bhattacherjee and Sanford [18], system experts are more inclined to critically appraise messaging to inform system attitudes and acceptance. They are more aware of the possibility of bias and inaccuracy in messaging, whereas users with lower levels of expertise tend to rely more on peripheral cues, rather than embedded message arguments, to form their opinions [18]. Thus, user expertise influences the extent of elaboration because it affects an individual’s ability to process information relating to the system [20,21].

### Motivation

#### Perceived Usefulness

Perceived usefulness is a function of productivity, performance, effectiveness, and overall usefulness of an object [18,22]. When there is a match between the user’s needs and what the source offers, the user’s motivation to process the message increases because it is deemed to be useful. Highly motivated users are more likely to elaborate on what the source is saying [23] and are more likely to distinguish between strong and weak arguments [19]. Strong arguments for these highly motivated individuals increase positive attitudes, whereas weak arguments result in a decrease in positive attitudes [19]. Those that perceive low usefulness will not expend the same effort on message elaboration as those that perceive high usefulness. In the context of health care, the perceived usefulness of technology has been shown to impact clinicians’ motivation to accept and use the technology [24].

#### Relevance to Job

Job relevance is defined by Bhattacherjee and Sanford [18] as the system’s relevance to the user’s work. To clarify the intended application of this term, our study used “relevance to job” to minimize confusion. Systems perceived to be highly relevant to one’s work tend to be subject to greater elaboration and scrutiny of related messaging. Users that perceive systems to have high relevance to their job are less likely to pay attention to peripheral cues, whereas those that perceive low relevance to job will not be motivated to dedicate effort to thoughtful processing and will rely on other, peripheral cues to shape their attitudes toward a system [18].

We examine how these antecedents affect attitudes toward, and intention to use, a VCoP system through the mediating routes of persuasion. Descriptions of the constructs used to represent these routes of persuasion are presented subsequently.

### Central Route of Persuasion (Argument Quality)

Quality arguments are those viewed as informative, helpful, valuable, and persuasive [18,21]. As documented in the extant literature [18,21,25], argument quality is used to represent the central route to persuasion. ELM considers argument quality to be the determining factor for whether information is influential when in a state of high elaboration [21]; argument quality has a greater impact on attitudes for those in a state of high elaboration than in low elaboration [26].

Our study presented high-quality arguments as six positively framed arguments (as listed in Table A1 in Multimedia Appendix 1), which Angst and Agarwal [14] have defined as those that contain both credible content and beneficial outcomes. Applied to a VCoP context, an example of such an argument could be:

Virtual communities of practice (VCoPs) have been shown to facilitate development of an innovative patient-focused integration of medical, social, and supportive services by health-care organizations, while allowing health-care providers to use their energy and time more efficiently and provide care that is collaborative and cost-effective.

6

The quality of the argument is found to be more persuasive under conditions of high relevance compared with low relevance [19]. When issues are perceived to be of high relevance, if arguments are strong, then increasing the number of arguments increases persuasion [19]. However, if the quality of the argument is weak, this reduces their persuasive effect [19]. In contrast, when issues are perceived to be of low relevance, more messages (regardless of their quality) serve as a peripheral cue signaling worthiness of the message and positively affect persuasion [19,26].

### Peripheral Route of Persuasion

#### Source Credibility

This construct refers to the perceived credibility of the message source but does not consider the message content itself [21,27]. Source credibility assesses the source’s knowledgeability, expertise, trustworthiness, and credibility [18,21]. It is considered a peripheral cue because source credibility is expected to be more important for those who are not experts [21]. For such individuals, the presence of source credibility could increase the favorability of an argument, and could even bias how the quality of the argument is perceived [21,28]. In a health care context, source credibility may be exhibited through a colleague’s opinion, a viewpoint from an opinion leader in the domain, or the organization affiliated with the product or system.

#### Connectedness

As a design element, connectedness is defined as the extent to which website visitors are able to express their views, benefit from the community of visitors to the website, and share a common bond with website visitors [25]. It is considered a peripheral cue because it does not pertain to the message content itself. Although classified and validated as a peripheral cue in a traditional information systems context [25], connectedness has not been investigated as a peripheral cue in a VCoP context. User satisfaction and knowledge self-efficacy (feeling knowledgeable and capable of helping others) have been shown to positively affect continued participation in VCoPs [29]; therefore, it is important that community members feel that their opinions matter and that they have influence on the group. Within CoPs, connection to other members and the value derived from them sustain the community’s activities [4]. Within a broader online context, a sense of connectedness with other participants or consumers has been shown to positively impact trust [30,31], willingness to return, and become loyal to the site [32-34]. These are necessary considerations for the continued usage of VCoPs.

### Research Question

Virtual CoPs are known to benefit health care workers [6,7,35-37]. However, the factors influencing their adoption of the technology to support them are not well understood [8,36]. Although past research has shown that the ELM’s central and peripheral routes influence attitude formation and change, and ultimately the intention to use an information system, our research seeks to gain insight into the potential antecedents of these two routes of processing among health care workers for VCoPs. To date, no research has investigated these precursors in this context. By understanding what influences these two persuasion routes, we can better understand how to design and position a virtual CoP for health care workers to encourage their use.

Thus, using ELM as a guiding framework, this research seeks to investigate the following question: For those working in health care, how does motivation and ability affect attitudes toward using VCoPs?

## Methods

### Sample and Procedure

Participants in this study were adults older than 18 years working in the health care system (either as a clinician, administrator, researcher, or nonclinical staff). This study aimed to determine how motivation and ability affect an individual’s attitudes toward health care technology adoption (specifically VCoPs); therefore, it was necessary that the participants worked in health care.

Surveys were administered at two large health care conferences that took place in Toronto during October 2016. The conferences were one day apart and had a total of approximately 3000 attendees who were predominantly health care providers, administrators, and academics. A confidence interval of 90% with a 10% margin of error was used to calculate a minimum sample size of 67 [38].

Data were collected using online surveys conducted on laptops at the conferences. Postcards (providing a link to the survey) also were handed out at the conferences for those who wished to complete the survey following the conference. There was no compensation for participation.

After an initial screening question to ensure that the participant worked in health care, each participant’s consent was obtained via a detailed consent form. They were then asked a series of questions regarding their knowledge of VCoPs and related technologies, and they were asked to evaluate a mock website interface of the home page and two subpages of a proposed virtual CoP. These mock-ups were used to provide participants with a sense of what a VCoP would look like and allow for visualization of our central and peripheral cues of investigation (argument quality, source credibility, and connectedness). Participants were then asked questions about their perceptions of the VCoP, which included argument quality (the strength of an informational message’s arguments), source credibility (the expertise of people providing endorsements), connectedness, and their attitude toward and intention to use such a system.

### Instrument

The survey contained 25 closed-ended questions, 3 open-ended questions, and 5 demographic questions. On average, it took participants approximately 15 minutes to complete the survey. To ensure content validity, measurement scales were selected from existing literature where they had been prevalidated. Some of the questions were slightly adapted to reflect the context of this study; all items were assessed using 7-point Likert scales for their respective questions. The full measurement instrument can be found in Multimedia Appendix 1. Based on previous research [18], the concept of ability was operationalized as user expertise across four areas (expertise with social networks, electronic medical records [EMRs], CoPs, and VCoPs). Motivation was represented through the constructs of relevance to job and perceived usefulness [18]. The two routes of persuasion are represented through argument quality (central route) and source credibility and connectedness (peripheral route) and were measured in alignment with extant ELM research [14,18,25]. Attitudes were assessed both at the beginning (initial attitude) and end (formed attitude) of the study to enable the examination of attitude change [14,18,39]. Finally, the intention to use such a platform was measured using a one-item scale (as per [18,39]).

The three open-ended questions probed further into understanding health care workers’ perceptions of VCoPs (“What would encourage you to use a VCoP?”; “What barriers would prevent you from using a VCoP?”; and “What helped you form your attitude toward the VCoP?”). Answers to these open-ended questions helped to provide insights to our interpretation of the closed-ended survey results. The demographic questions (age, gender, job title, sector, and education) were used to ensure our sample was representative of the broader health care worker population and to examine potential effects on the primary constructs of interest.

### Analysis Strategy

To examine the proposed research model, the following steps were conducted: (1) the direct effects of user characteristics (ability and motivation) were examined on outcome variables (formed attitude, attitude change, and intention to use); (2) user characteristic constructs were divided into high- and low-level groups (ie, high and low levels of ability or expertise as well as motivation) and these groups were examined for their effects on outcome variables; and (3) based on the findings from step 2, high- and low-level groups of user characteristics were examined across information processing routes (central and peripheral) for their effects on the outcome variables.

Spearman rho correlations were used to test relationships between variables in the research model. The decision to use a nonparametric test was made because the outcomes of interest were found to violate the assumption of normality when using Shapiro-Wilk normality. This research introduced a new health care context to apply the ELM to investigate attitude formation toward VCoP; therefore, correlation was deemed an appropriate test for examining what, if any, potential relationships existed between the antecedents, persuasion routes, and outcomes.

The antecedent constructs for ability (user expertise) and motivation (relevance to job and perceived usefulness) were divided into higher (≥5 on a 7-point scale) and lower (≤3 on a 7-point scale) user groups. Answers of 4 (on a 7-point scale) were not included in this analysis to create a distinct separation of user groups for the analysis and to increase the rigorousness of the analysis.

Data were analyzed using JGR version 1.7-16 statistical software, which is an open-source graphical user interface for R. The total number of participants for the online survey was 88, from which two respondents were removed for insufficient responses to questions. This exceeded our minimum sample size requirement of 67.

## Results

### Participant Characteristics

Full demographic information of the participants is provided in Table 1. The mean age of participants was 39.98 (SD 10.84) years**,** reflective of the average age (ie, 43) of the health care workforce [40]. Females made up 70% (58/86) of the sample. According to the Canadian Institute for Health Information, 80% of Canadian health care workers are female [40].

**Table 1:** Characteristics of the participants who completed the online survey (N=86).

Characteristic		Participants
Age (years; N=76), mean (SD)		39.98 (10.84)
**Gender (N=83), n (%)**		
	Female	58 (70)
	Male	25 (30)
**Primary job title, n (%)**		
	Physician	5 (6)
	Nurse	8 (9)
	Allied health professional	3 (4)
	Administrator	26 (30)
	Nonclinical staff	9 (10)
	Researcher	3 (4)
	Student	3 (4)
	Other	27 (31)
	Unknown^a^	2 (2)
**Primary sector, n (%)**		
	Academia	3 (4)
	Association	1 (1)
	Community mental health and addictions	2 (2)
	Government	20 (23)
	Home and community care	6 (7)
	Hospital	23 (27)
	Industry	2 (2)
	Long-term care	9 (10)
	Primary care	7 (8)
	Other	11 (13)
	Unknown^a^	2 (2)
**Education, n (%)**		
	Some high school or less	0 (0)
	Completed high school or GED	0 (0)
	Some college	0 (0)
	College diploma	3 (4)
	Undergrad or bachelor’s degree	27 (31)
	Master’s degree	39 (45)
	Beyond master’s	10 (12)
	Other^b^	5 (6)
	Unknown^a^	2 (2)

^a^Unknown: participant did not provide answer.

^b^Other: PhD (n=2); current master’s student (n=1); postgraduate master’s certificate (n=1); MD, CCFP, FCF (n=1).

### Scale Validation

With the exception of user expertise and intention to use, all constructs in this research are reflective in nature. They were each measured by multiple survey questions (items) in which these items were expected to correlate with one another and share a common theme. User expertise probed into self-reported experience with technologies and forums relevant to this investigation and was not necessarily expected to correlate. Intention to use was measured using a single item adapted from Bhattacherjee and Sanford [18]. To measure the internal consistency of the reflective constructs in our survey instrument, Cronbach alphas were assessed, which all met the recommended threshold of 0.70 [41]. Multimedia Appendix 2 provides details of our scale validation.

### Key Findings

Table 2 provides the results of the first step of our analysis in which the direct effects of user characteristics (ability and motivation) were examined on outcome variables (attitude change, formed attitude, and intention to use). Both motivation constructs of perceived usefulness and relevance to job were significantly correlated with formed attitude and intention to use (*P*<.001). Unlike the user expertise construct, there were no significant correlations with motivational constructs and attitude change.

**Table 2:** Direct influence of user characteristic antecedents on outcomes (N=86).

Characteristic		Attitude change		Formed attitude		Intention to use	
		Spearman rho	*P* value	Spearman rho	*P* value	Spearman rho	*P* value
**User expertise**							
	Online social networks	−0.104	.34	0.112	.30	0.164	.13
	Electronic medical records	−0.060	.58	−0.004	.97	0.076	.49
	Communities of practice (CoPs)	−0.215	.047	0.165	.13	0.362	.001
	Virtual communities of practice (VCoPs)	−0.222	.04	0.068	.53	0.279	.009
Perceived usefulness		0.022	.84	0.349	.001	0.512	.001
Relevance to job		0.000	.99	0.385	.001	0.428	.001

The second step of our analysis separated both ability and motivation user characteristics into higher- and lower-level groups (higher group: >4 on 7-point Likert scale; lower group: <4 on 7-point Likert scale) to examine for their effects on outcome variables. For the motivation user characteristics (relevance to job and perceived usefulness constructs), the vast majority of participants fell into the higher category. Similarly, most participants fell into the higher category for their familiarity with online social networks and EMRs. Thus, meaningful comparisons across high and low levels of these motivation constructs and the first two experience items were not possible. However, CoPs and VCoPs expertise did reveal differences between low- and high-expertise groups. Higher expertise users for both CoPs and VCoPs exhibited greater intention to use a health care VCoP than those with lower expertise (*F*_1,56_=7.800, *P*=.007; *F*_1,67_=6.199, *P*=.02, respectively). When examining high and low group differences for attitude change, expertise with CoPs stood out in terms of its significance (*F*_1,54_=5.006, *P*=.03) between higher- and lower-level experience groups (n=45 and n=30, respectively).

The third step of our analysis involved delving more deeply into the significant results of the previous step by examining high- and low-level groups of user characteristics (specifically, CoPs expertise) across information processing routes (central and peripheral) for their effects on outcome variables. As shown in Table 3, central route cues (operationalized as argument quality) were the most important persuasion route for both those with higher and lower levels of expertise. Those with higher levels of CoPs expertise were also influenced by peripheral cues of source credibility (*P*=.005 for formed attitude and intention to use the system) and connectedness (*P*=.04 for formed attitude; *P*=.008 for intention to use the system), whereas those with lower levels of CoPs expertise were not (*P*>.05).

**Table 3:** Impact of persuasion routes on outcomes by expertise level (N=75).

Expertise level		Argument quality		Source credibility		Connectedness		Formed attitude	
		Spearman rho	*P* value	Spearman rho	*P* value	Spearman rho	*P* value	Spearman rho	*P* value
**Lower (n=30)**									
	Attitude change	−0.233	.22	0.131	.49	0.214	.26	0.237	.21
	Formed attitude	0.377	.04	0.109	.56	0.189	.32		
	Intention to use	0.413	.02	0.118	.53	0.203	.28	0.187	.32
**Higher (n=45)**									
	Attitude change	0.193	.20	0.193	.20	0.232	.12	0.290	.05
	Formed attitude	0.433	.003	0.412	.005	0.314	.04		
	Intention to use	0.440	.003	0.416	.005	0.392	.008	0.554	<.001

## Discussion

### Principal Results

Using the ELM framework, this research found that both ability and motivation play an important and positive role in the adoption of health care VCoPs. Health care workers that perceived the VCoP to be useful and relevant to their job (motivation constructs) had a significantly more positive attitude formation toward and intention to use the system. These motivation constructs were more strongly (positively) correlated with argument quality than they were with the peripheral cues of source credibility and connectedness.

For user expertise (ability construct), familiarity with online social networks and EMRs did not play a role in the perceptions and adoption of a VCoP; however, experience with CoPs and VCoPs both had statistically significant negative correlations with attitude change. This means that the higher the experience level, the smaller the change in attitude after experiencing our experimental VCoP. Conversely, the lower the experience level with CoPs and VCoPs, the larger the change in attitude.

Health care workers with higher CoPs and VCoPs expertise exhibited greater intention to use a health care VCoP than those with lower expertise. As expected, these higher and lower levels of expertise groups differed in their processing routes. Although the central processing route (operationalized as argument quality) was the most important persuasion route for both levels of CoPs expertise, those with higher levels of expertise were also influenced by peripheral cues of source credibility and connectedness.

### Connections to Previous Work

To date, the application of ELM to the field of eHealth remains limited and has been from the patient perspective and not the provider [14]. This study was the first to apply ELM to investigate attitude formation toward VCoPs for those working in health care and quality improvement. Although connectedness has been investigated as an ELM peripheral cue for traditional information systems [25], our study is the first to include this construct when investigating VCoPs in health care. Additionally, the investigation of antecedents to the validated concepts of central and peripheral routes to persuasion for health care VCoPs is novel. Thus, when compared with extant research on health care VCoPs, this study presents a unique population (health care providers) and provides insights through new constructs of investigation in this context (ability and motivation antecedents as well as the connectedness construct).

The results of this study confirmed previous ELM work that showed the importance of central and peripheral routes of persuasion for attitude formation or change and intention to use ICTs [13,14,18,29]. The importance of argument quality as a central route to persuasion was validated [18,21,25]. Surprisingly, argument quality, traditionally found most effective in persuading those in states of high elaboration [21,26], was found to be an effective influencer on attitude formation for both those with higher and lower levels of CoP expertise (ie, both high and low elaboration states).

Another interesting and surprising result related to expertise (specifically CoP expertise) was that attitude formation of those with low levels of CoP user expertise was most influenced by central rather than peripheral routes of persuasion. Connectedness and source credibility, traditionally seen as peripheral cues [18,25], were only significantly correlated with formed attitude for those with high levels of CoP user expertise. The most important factor contributing to positive attitudes for those with low levels of CoP user expertise was the central route, operationalized as argument quality. This conflicts with extant ELM research in non-health care contexts that found peripheral cues to most influence those in states of lower elaboration. Specifically, previous work has found that users with lower levels of system expertise tend to rely more on peripheral cues, rather than embedded message arguments, to form their opinions [18].

### Implications

Rolls et al [9] found that those working in health care see virtual communities as a source for relevant clinical information to inform their clinical decision making, especially given the limited communication channels, which are a known barrier to translating research into practice. The potential for such systems to improve health care quality has been demonstrated, but little is known about how and why health care workers adopt technologies to support CoPs [9-11]. This research helps us to understand the factors influencing the use of health care VCoPs, thus providing a basis for understanding how to best design and position such systems to encourage their effective use among health care workers.

Our research showed that health care user motivation (perceived usefulness and relevance to job) has a positive impact on one’s formed attitude and intention to use health care VCoPs. Although there were no significant correlations between motivational constructs and attitude change, this is not surprising given the overall high rating of these two constructs in our sample. The health care workers in our study were already highly motivated (saw VCoPs as being useful and relevant to their jobs); therefore, there were no significant differences between pre and post measures of attitude. The association of high motivation with strong attitudes and intention to use highlights the importance of these factors in determining the likelihood of the technology’s success in a health care context. The implication is that manipulation of these factors, such as through leadership and organizational support to socialize and normalize the value of CoPs and VCoPs (for relevance to job and perceived usefulness), may offer a promising approach to improve their likelihood of adoption.

For ability (specifically, experience with CoPs and VCoPs), our research showed that this factor directly affects attitude change or intention to use VCoPs. Unlike motivation constructs, there was a broad range of prior expertise with CoPs and VCoPs in our sample. As our results show, health care workers with less prior experience with such systems experience a significantly positive attitude change toward these systems through initial exposure. Thus, this initial exposure to gain familiarity with VCoPs is an important opportunity for practitioners to establish positive attitudes toward such systems among health care workers. Further, the strong correlation found between attitudes and intention to use the system for those with higher levels of expertise highlights the importance of understanding how to change attitudes, and thus intention to use, among those with lower levels of expertise.

Although experience with CoPs and VCoPs provides interesting insights in establishing positive attitudes for health care VCoPs, the other two ability items (expertise with online social networks and EMRs) did not reveal any significant correlations. One explanation for this result may be that these particular items were not as applicable to this context given their high level of experience and expertise with the health care sample. These two items could not be separated into high and low expertise levels because everyone was highly familiar with these systems. Online social networks have now become commonplace; therefore, their relevance or ability to impact attitudes for other types of systems has diminished. If everyone is highly experienced with this technology, it may no longer be an effective predictor for acceptance of another technology. Although EMRs are a more specific technology, the same could be argued for its understanding and skillfulness among health care professionals. All health care professionals in our sample were highly familiar with EMRs; therefore, it no longer proved to be a predictor for acceptance of VCoPs. The implication is that these two expertise items may no longer be relevant to today’s health care professionals in examining VCoPs technology acceptance.

Although the findings about the processing routes by level of expertise run counter to ELM theory, the highly educated participants and the nature of their work are reflective of an evidence-minded study population. Given the importance of evidence for health care, these research findings are not surprising but do hold important clues for how to effectively appeal to different levels of user expertise. Rather than using source credibility and connectedness for those yet to experience the system, providing evidence of the system’s effectiveness is a promising tactic to use. Once persuaded to use the system, other messaging cues, such as the credibility of the source and the interface’s sense of connectedness, can be used to further entrench attitudes and strengthen the intention to use. Through the insights gained about the unique antecedents to attitude formation in health care, the design and messaging can be improved to appeal to those experienced with CoPs in their traditional offline form to translate this experience to usage of an online environment. For both those with higher and lower levels of expertise, evidence-based arguments are indeed an effective messaging tactic to improving attitudes toward VCoPs.

Given the novelty of VCoPs for quality improvement in health care in Canada, this research provides needed insight into effective messaging to increase the technology’s uptake and use. The relative newness of this technology for health care means opinions are not long-held; therefore, exposure to related information determines attitudes regarding its usage. To derive the maximum benefit from existing government expenditure and investment in eHealth, it is crucial to capitalize on this period when attitudes are being formed to create positive attitude changes. Increasing the capacity for sharing of evidence-based knowledge to support its implementation requires changes to structures and processes, which VCoPs have been shown to address effectively. Thus, improving the usage and adoption of VCoPs warrants investigation of the factors influencing attitudes toward such platforms. This study has provided insight into the antecedents to attitude formation, and the differences in influential factors between those with higher and lower levels of expertise.

### Limitations and Future Research Directions

It is acknowledged that the conferences at which this study’s surveys were administered may have biased the sample. Given that the conference participants were already actively involved in health care quality improvement by nature of their attendance, they may already have been “converted” to such concepts as CoPs. However, as this represents the target audience for such a technology, these research findings can have greater contextual relevance.

Other potential limitations are the environment in which the surveys took place and the length of the survey. Survey booths were located in high-traffic areas, and the majority of participants completed the survey during break periods; therefore, the potential for distraction as an external influence is recognized.

Another limitation is that this investigation was conducted in a Canadian context, in which health care is predominantly public based. Thus, these results may not be immediately transferrable to countries that have different health care systems or different socioeconomic, demographic, or cultural characteristics.

This study provides an important stepping stone to understand how attitudes of health care workers are formed for virtual communities of practice. There are several opportunities for future research to help deepen our understanding and further generalize our results. First, there is an opportunity to broaden the sample across different venues and contexts of health care. Venues that target different types of health care workers may allow for some interesting comparisons across professions (for example, physicians versus nurses versus administrators). Sampling across health care systems may also reveal some insights unique to public and private systems. Second, this study focused on the preusage stage of VCoPs, seeking to understand the factors that influence attitudes toward and intention to use such systems. Although participants had an opportunity to view a system mock-up from which they based their preusage survey responses, a longitudinal investigation of actual VCoP interaction may reveal insights to encourage continued usage. Finally, there are several other antecedents that could impact persuasion routes of attitude formation and change for VCoPs. This study focused on user experience, perceived usefulness, and relevance to job as ability and motivation factors. Research in non-health care contexts has shown that privacy, social influence, argument framing, and individual characteristics (age, gender, personality) may impact attitude formation or change and intention to use such systems. Future studies can explore the potential impact of these other types of variables.

### Conclusion

Although ELM’s framing of the central and peripheral routes to persuasion have been shown to influence attitude formation and change, and ultimately intention to use, this research gathered insights into potential antecedents of these two routes. By understanding what influences these two routes, we can better understand how to design and position a virtual CoP for health care practitioners.

There are challenges in bringing evidence into practice in health care [42]; however, this research highlights an exciting opportunity to translate research findings through peer knowledge-sharing in a trusting, online environment. Showcasing VCoPs value to members through the interface and eventual effects on practice will be a necessary future endeavor to ensure continued usage, and warrants further investigation. Health care resources are increasingly stretched. Enabling effective collaboration through VCoPs will contribute positively to fostering a culture of health care quality improvement.

## Appendix

Multimedia Appendix 1 Survey instrument.

Multimedia Appendix 2 Scale validation.

## Abbreviations

CoP: community of practice
EHR: electronic health record
ELM: elaboration likelihood model
ICT: information and communications technology
VCoP: virtual community of practice
