# Supplementary material for: Attitudes Toward Health Care Virtual Communities of Practice: Survey Among Health Care Workers
Source: J Med Internet Res. 2019 Dec 4;21(12):e15176. doi: 10.2196/15176 (PMC6920901; doi:10.2196/15176)
Supplement: Multimedia Appendix 1 [file jmir_v21i12e15176_app1.docx]

# Multimedia Appendix 1 – Survey Instrument

**Table A1.** Operationalization of constructs.

| Construct | Definition and/or source | Items |
| --- | --- | --- |
| **User expertise**^a^ | The ability of the participant to use the system in general [1]. | How knowledgeable are you on using the following technologies/ concepts?  1. Online social networks  2. Electronic medical records  3. Communities of practice (CoP) (in-person or offline)  4. Virtual communities of practice (VCoPs) |
| **Relevance to job**^b^ | The system’s relevance to the user’s work (Bhattacherjee & Sanford [1], citing Venkatesh & Davis [2]) | 1. Using a virtual CoP is important for my job.  2. Using a virtual CoP is relevant (appropriate) for my job. |
| **Perceived usefulness** | A function of productivity, performance, effectiveness and overall usefulness of an object (Bhattacherjee & Sanford [1], citing Davis, Bagozzi, & Warshaw [3]). | 1. Participating in a virtual CoP in my job will increase my productivity.  2. Participating in a virtual CoP in my job will improve my performance.  3. Participating in a virtual CoP will make me more effective (e.g. help me make better decisions).  4. I find VCoPs to be useful in my job. |
| **Connectedness** | Enabling visitors to share opinions, benefit from others from other visitors and share common interests with others [4] | 1. Visitors to this website share their views about quality improvement with other visitors of this website.  2. Visitors to this website benefit from the community visiting the website.  3. Visitors to this website share a common bond with other members of the community visiting the website. |
| **Argument quality** | Presented as six positively-framed arguments, which Angst & Agarwal [5] define as those that contain both credible content and beneficial outcomes. (Bhattacherjee & Sanford [1], referencing the Sussman & Siegal [6] evaluation of argument quality as informative, helpful, valuable, and persuasive.) | Please read the following statements:   1. “CoPs can have a role in achieving a diverse range of outcomes including, but not limited to, gaining competencies following completion of basic training; breaking down professional, geographical and organizational barriers; sharing information; reducing professional isolation; and facilitating the implementation of new processes and technology.” – BMC Health Services Research, 2011 [7] 2. Virtual communities of practice (VCoPs) have been shown to facilitate development of an innovative patient-focused integration of medical, social and supportive services by health-care organizations, while allowing health-care providers to use their energy and time more efficiently and provide care that is collaborative and cost-effective. – Health Expectations, 2003 [8] 3. Those working in health care organizations are able to access organizational knowledge through the use of VCoPs on an as-needed basis, incorporate it into their daily work and convert their new knowledge to future health care needs at the organization. – Health Expectations, 2003 [8] 4. Studies reveal that VCoPs enable health care teams to “collaborate in order to share, debate, resolve, integrate and implement different perspectives on practice to improve and inform evidence-based decision making.” – Social Networking, 2016 [9] 5. VCoPs allow for extension of learning in health care beyond face-to-face opportunities through the promotion of distributed and continued learning. – Technology Enabled Knowledge Translation for eHealth, 2012 [10] 6. VCoPs strengthen intra-professional ties, improve access to information and provide support otherwise unavailable to health care practitioners. – Journal of Advanced Nursing, 2011 [11]   The information provided about VCoPs was informative.  The information provided about VCoPs was helpful.  The information provided about VCoPs was valuable.  The information provided about VCoPs was persuasive. |
| **Neutral argument framing / source credibility** | The perceived credibility of the source but does not consider the message itself [6,12]. Assesses the source’s knowledgeability, expertise, trustworthiness and credibility [1,6]. Four neutrally-framed arguments were used as done by Angst & Agarwal [5]. | Please read the following statements:   1. Health Quality Ontario is launching a VCoP 2. “Quality improvement professionals recognize the value of virtual communities of practice.” – VP of Quality Improvement at Health Quality Ontario 3. “[C]ommunities of practice (CoPs) have become increasingly popular within the health sector.” – Implementation Science, 2009 [13] 4. “My CoP influences the care I deliver.” – Dr. Noah Ivers (MD, PhD, CCFP), Family Physician and Scientist at Women’s College Hospital   The sources of information demonstrate knowledge.  The sources of information are trustworthy.  The sources of information are credible.  The sources of information appear to be experts on topic. |
| **Pre/post attitudes**^c,d^ | Angst & Agarwal [5] and Bhattacherjee & Sanford [1]; the latter used the Taylor & Todd [10] semantic differential scale of adjective pairs. | With what you (now)^d^ know about VCoPs, please answer the following question. What are your feelings about VCoPs for health care quality improvement?  1. Bad to Good  2. Foolish to Wise  3. Unimportant to Important |
| **Intention to use** | Bhattacherjee & Sanford [1], referencing the Taylor & Todd [14] Likert scale. | I intend to use the Online Environment or other virtual community of practice in the next 3–6 months. |

^a^ Rated on a 7-point scale of novice to expert.

^b^ To clarify the intended application, the current research used “Relevance to job” rather than “Job relevance.”

^c^ Rated on a 7-point scale for each of Bad to Good, Foolish to Wise, and Unimportant to Important.

^d^ The statement with the word “now” was only assessed when measuring post attitudes.

**References**

1. Bhattacherjee A, Sanford C. Influence processes for information technology acceptance: An elaboration likelihood model. MIS Quarterly 2006 Dec 1:805-25. doi: 10.2307/25148755
2. Venkatesh V, Davis FD. A theoretical extension of the technology acceptance model: Four longitudinal field studies. Manag Sci 2000 Feb;46(2):186-204. doi: 10.1287/mnsc.46.2.186.11926
3. Davis FD, Bagozzi RP, Warshaw PR. User acceptance of computer technology: a comparison of two theoretical models. Management Sci 1989 Aug;35(8):982-1003. doi: 10.1287/mnsc.35.8.982
4. Cyr D, Head M, Lim E & Stibe A. The art of online persuasion through design: The role of issue involvement as it influences users based on prior knowledge. In Proceedings for the Fourteenth Annual Workshop on HCI Research in MIS (HCI/MIS’12); 2015. Fort Worth, Texas.
5. Angst CM, Agarwal R. Adoption of electronic health records in the presence of privacy concerns: The elaboration likelihood model and individual persuasion. MIS Quarterly 2009 Jun 1;33(2):339-70. doi: 10.2307/25148755
6. Sussman SW, Siegal WS. Informational influence in organizations: An integrated approach to knowledge adoption. Inform Syst Res 2003 Mar;14(1):47-65. doi: 10.1287/isre.14.1.47.14767
7. Ranmuthugala G, Plumb JJ, Cunningham FC, Georgiou A, Westbrook JI, Braithwaite J. How and why are communities of practice established in the health care sector? A systematic review of the literature. BMC Health Serv Res 2011 Dec;11(1):273. doi: 10.1186/1472-6963-11-273
8. Winkelman WJ, Choo CW. Provider‐sponsored virtual communities for chronic patients: improving health outcomes through organizational patient‐centred knowledge management. Health Expect 2003 Dec;6(4):352-8. doi: 10.1046/j.1369-7625.2003.00237.x
9. Ikioda F, Kendall S, Brooks F, De Liddo A, Shum SB. Factors That Influence Health care Professionals’ Online Interaction in a Virtual Community of Practice. Social Networking 2013 Oct 30;2(4):174-84. doi:10.4236/sn.2013.24017
10. Jarvis-Selinger S, Armstrong A, Mehta S, Campion E, Black K. Orthopaedic Educators’ Electronic Community of Practice: Development of a Supportive Online Learning Environment for Academic Orthopedic Surgeons. In K. Ho, S. Jarvis-Selinger, H. Novak Lauscher, J. Cordeiro, & R. Scott (Eds.), Technology Enabled Knowledge Translation for eHealth; 2012:117-31.
11. Valaitis RK, Akhtar‐Danesh N, Brooks F, Binks S, Semogas D. Online communities of practice as a communication resource for community health nurses working with homeless persons. J Adv Nurs 2011 Jun;67(6):1273-84. doi: 10.1111/j.1365-2648.2010.05582.x
12. Chaiken S. Heuristic versus systematic information processing and the use of source versus message cues in persuasion. J Pers Soc Psychol 1980 Nov;39(5):752-76.
13. Li LC, Grimshaw JM, Nielsen C, Judd M, Coyte PC, Graham ID. Use of communities of practice in business and health care sectors: a systematic review. Implement Sci 2009 Dec;4(1):27. doi: 10.1186/1748-5908-4-27
14. Taylor S, Todd PA. Understanding information technology usage: A test of competing models. Inf Sys Res 1995 Jun;6(2):144-76. doi: 10.1287/isre.6.2.144
