# Supplementary material for: Attitudes Toward Health Care Virtual Communities of Practice: Survey Among Health Care Workers
Source: J Med Internet Res. 2019 Dec 4;21(12):e15176. doi: 10.2196/15176 (PMC6920901; doi:10.2196/15176)
Supplement: Multimedia Appendix 2 [file jmir_v21i12e15176_app2.docx]

# Multimedia Appendix 2 – Scale Validation

Cronbach’s alpha values calculate the correlations between individual survey items for the corresponding reflective construct. Values above 0.70 are considered acceptable, with those above 0.80 considered excellent. If the values are lower than the acceptable threshold, this may indicate other unintended characteristics are being measured [1]. As shown in table A2, all survey items had Cronbach’s alpha values above the recommended 0.70 level.

**Table A2.** Cronbach’s alpha results for survey items.

| Construct | Cronbach’s alpha | Mean (SD) |
| --- | --- | --- |
| Argument quality | 0.91 | 5.6 (0.92) |
| Source credibility | 0.87 | 5.6 (0.90) |
| Connectedness | 0.77 | 5.7 (0.79) |
| Relevance to job | 0.89 | 5.5 (1.0) |
| Perceived usefulness | 0.92 | 5.3 (1.0) |
| Attitude | 0.90 | 6.2 (0.72) |

**References**

1. Hulley SB, Cummings SR, Browner WS, Grady DG, Newman TB. Designing clinical research. 3rd edition. Lippincott Williams & Wilkins, Philadelphia;2007. p.249. ISBN-13: 978-0-7817-8210-4
